# Effect of everolimus on the glucose metabolic pathway in mouse skeletal muscle cells (C2C12)

**DOI:** 10.1007/s11306-017-1236-5

**Published:** 2017-07-07

**Authors:** Kayoko Yoshida, Chiyo K. Imamura, Kanako Hara, Mayumi Mochizuki, Yusuke Tanigawara

**Affiliations:** 1 Department of Clinical Pharmacokinetics and Pharmacodynamics, Keio University School of Medicine, 35 Shinanomachi, Shinjuku-ku, Tokyo 160-8582 Japan; 2 Division for Evaluation and Analysis of Drug Information, Faculty of Pharmacy, Keio University, Minato-ku, Tokyo Japan

**Keywords:** mTOR inhibitor, Everolimus, Hyperglycemia, Glycometabolism, Metabolomics, CE-TOFMS

## Abstract

**Introduction:**

Everolimus selectively inhibits mammalian target of rapamycin complex 1 (mTORC1) and exerts an antineoplastic effect. Metabolic disturbance has emerged as a common and unique side effect of everolimus.

**Objectives:**

We used targeted metabolomic analysis to investigate the effects of everolimus on the intracellular glycometabolic pathway.

**Methods:**

Mouse skeletal muscle cells (C2C12) were exposed to everolimus for 48 h, and changes in intracellular metabolites were determined by capillary electrophoresis time-of-flight mass spectrometry. mRNA abundance, protein expression and activity were measured for enzymes involved in glycometabolism and related pathways.

**Results:**

Both extracellular and intracellular glucose levels increased with exposure to everolimus. Most intracellular glycometabolites were decreased by everolimus, including those involved in glycolysis and the pentose phosphate pathway, whereas no changes were observed in the tricarboxylic acid cycle. Everolimus suppressed mRNA expression of enzymes related to glycolysis, downstream of mTOR signaling enzymes and adenosine 5′-monophosphate protein kinases. The activity of key enzymes involved in glycolysis and the pentose phosphate pathway were decreased by everolimus. These results show that everolimus impairs glucose utilization in intracellular metabolism.

**Conclusions:**

The present metabolomic analysis indicates that everolimus impairs glucose metabolism in muscle cells by lowering the activities of glycolysis and the pentose phosphate pathway.

**Electronic supplementary material:**

The online version of this article (doi:10.1007/s11306-017-1236-5) contains supplementary material, which is available to authorized users.

## Introduction

Everolimus is a molecular-targeted agent used in oncology and organ transplantation. It binds to the FK506 binding protein FKBP12 and generates potent inhibition of mammalian target of rapamycin complex 1 (mTORC1), which lies downstream of the phosphatidylinositol 3-kinase (PI3K)/Akt pathway (Kirchner et al. 2004). In mammalian cells, mTOR protein has two forms that have different structures and functions: mTORC1 and mTORC2. These mTOR proteins function as signal-transducing proteins. The acceleration and regulation translational factors ribosomal protein S6 kinases 1 (S6K1) and eukaryotic translation initiation factor 4E-binding protein 1 (4EBP1), and the transcription factors hypoxia-inducible factor-1α (HIF1α) and c-Myc are located downstream of mTOR (Cheng et al. 2014; Hay and Sonenberg 2004; Wullschleger et al. 2006; Yeung et al. 2008).

mTORC1 activates translation vital for survival by intensifying S6K1 and suppressing 4EBP1 activity. Several neoplastic cells have abnormal PI3K/Akt signaling, which may cause excessive activation of mTOR proteins. Everolimus selectively inhibits mTORC1, blocks cell growth and proliferation, and is used in oncology settings, such as the treatment of breast cancer, neuroendocrine tumors, renal cell carcinoma (RCC), and subependymal giant cell astrocytoma with renal angiomyolipoma or tuberous sclerosis complex (Chiang and Abraham 2007; Shaw and Cantley 2006).

Metabolic toxicities have emerged as a common and unique side effect of mTOR inhibitors. Everolimus induces hyperglycemia in 10–50% of all patients, with approximately 10% graded as severe (Baselga et al. 2012; Bissler et al. 2013; Franz et al. 2013; Motzer et al. 2008; Sivendran et al. 2014; Yao et al. 2011). Hyperglycemia is one of the major side effects but is less frequent when everolimus is used as an immunosuppressant than an anticancer drug (Lorber et al. 2005; Vitko et al. 2004, 2005), probably because a lower dose is used as an immunosuppressant. Another mTOR inhibitor, temsirolimus, which is used intravenously to treat RCC, causes hyperglycemia in approximately 50% of patients (Hudes et al. 2007; Sivendran et al. 2014).

Previous studies have reported that rapamycin, an mTOR inhibitor, may cause hyperglycemia by inhibiting insulin secretion or insulin resistance (Lamming et al. 2012; Rachdi et al. 2008). Because everolimus selectively inhibits mTORC1 and because mTORC1 resides in cells throughout the body, it is also important to consider perturbation of glucose utilization in peripheral tissues in addition to insulin-related mechanisms when discussing the pathogenesis of hyperglycemia attributed to everolimus. However, this viewpoint has not previously been reviewed in the literature. Therefore, in this study, we examined the effect of everolimus on intracellular glycometabolism using mouse skeletal muscle cells (C2C12), which are commonly used in basic studies of diabetes, with a novel comprehensive metabolomic analysis method. Comprehensive metabolomic analysis is a breakthrough method capable of clarifying the global profiles of abundant metabolites. In particular, capillary electrophoresis time-of-flight mass spectrometry (CE-TOFMS) is suitable for analysis of ionic metabolites such as those found in glucose metabolism (Soga et al. 2003). In this article, we focus on the three major glucose metabolic pathways: glycolysis, the tricarboxylic acid (TCA) cycle, and the pentose phosphate pathway (PPP), and report a new mode of action of everolimus on glycometabolism in C2C12 cells.

## Materials and methods

### Chemicals and reagents

Everolimus (RAD001) was obtained from Selleck Chemicals (Houston, TX, USA). Ascomycin was obtained from LC-Laboratories (Woburn, MA, USA). d(+) glucose and Dulbecco’s modified Eagle’s medium (DMEM) were purchased from Wako Pure Chemical Industries, Ltd. (Chuo-ku, Osaka, Japan). Fetal bovine serum (FBS) was purchased from Life Technologies (Carlsbad, CA, USA). Internal Standard Solution-1 and Internal Standard Solution-3 were obtained from Human Metabolome Technologies, Inc. (HMT; Tsuruoka, Yamagata, Japan). Methanol, chloroform, and acetonitrile were high-performance liquid chromatography (HPLC) grade, and water was distilled and deionized (Milli-Q). Polyclonal anti-glucose transporter 1 (GLUT1) antibody (07-1401) was purchased from Merck Millipore (Darmstadt, Germany). Hexokinase (HK)-1 (C35C4) rabbit monoclonal antibody (mAb) (#2024), pyruvate kinase (PKM1/2) (C103A3) rabbit mAb (#3190), pyruvate kinase (PKM2) (D78A4) XP^®^ rabbit mAb (#4053), and lactate dehydrogenase A (LDHA) antibody (#2012) were purchased from Cell Signaling Technology (Danvers, MA, USA). Mouse phosphofructokinase (PFKM) antibody (MAB7687) was purchased from R&D systems (Minneapolis, MN, USA). Anti-phosphogluconate dehydrogenase (PGD) antibody [EPR6565] (ab129199) was purchased from Abcam (Cambridge, UK). Transaldolase (TALDO) (H-4) mouse monoclonal immunoglobulin G was purchased from Santa Cruz (Dallas, TX, USA). Monoclonal anti-γ-tubulin primary antibody (T6557) was purchased from Sigma-Aldrich (St. Louis, MO, USA).

### Cell culture and everolimus exposure

C2C12 mouse myoblasts were obtained from RIKEN Cell Bank (Tsukuba, Ibaraki, Japan). C2C12 mouse myoblasts (1 × 10^5^ cells) were seeded into a 6-cm dish and cultured in DMEM supplemented with 10% FBS for metabolomics analysis and Western blotting. For real-time reverse transcription-polymerase chain reaction (real-time RT-PCR) and enzyme activity assays, 5 × 10^2^ C2C12 mouse myoblast cells were seeded into each well of a 96-well plate and cultured in DMEM supplemented with 10% FBS. Differentiation of C2C12 cells was induced by switching confluent cells to a differentiation medium (DMEM with 2% FBS) and allowing formation of myotubes, with media changes every 24 h. Everolimus was dissolved in methanol and diluted with a differentiation medium to 500 ng/mL. The C2C12 myotubes were exposed to either 4 mL or 100 µL of everolimus-medium solution in each dish or well of a 96-well plate, respectively. Control cells were incubated in a differentiation medium containing 0.01% methanol. To exclude non-specific toxicities, we assessed cell viability in both treated and untreated cells. After 48 h, the cell viability of everolimus-exposed cells was 91.3% and that of control cells was 91.0%. There was no significant difference of cell viability between everolimus-treated and untreated cells.

### Sample preparation

Preliminary experiments with different exposure times showed that 48 h exposure was optimal. After 48 h of everolimus treatment, 100 µL of the culture medium in the dish was collected to measure the glucose concentration. The sample preparations in this study were modified using a previously reported method (Makinoshita et al. 2014; Soga et al. 2003). Remaining culture medium was removed from the dish using an aspirator. Cells were washed twice with ice cold 5% mannitol solution and dispersed for 10 min in 1.4 mL of ice cold methanol containing 20 µg/mL of ascomycin used as an internal standard for everolimus determination, and 10 µM Internal Standard Solution-1 used as an internal standard for metabolomic analysis. The solution was centrifuged (4 °C, 1000×*g*, 10 min), and 980 µL of supernatant was added to a tube. Next, 20 µL of methanol, 400 µL of water, and 1000 µL of chloroform were added and mixed well. The mixture was centrifuged (4 °C, 4600×*g*, 10 min). The upper layer (aqueous layer) was used for glucose assay and metabolomics analysis. The lower layer (organic layer) was used for everolimus determination.

### Determination of everolimus taken up by C2C12 myotube cells

A 750 µL aliquot of the organic layer of C2C12 cell extract was evaporated to dryness under air at 40 °C. The dried residue was reconstituted in 500 µL of mobile phase solution and transferred to an autosampler vial. The concentration of everolimus was determined using an HPLC-ultra violet (UV) method developed specifically for this study by modification of a previously reported method (Khoschsorur 2005). A Waters 2695 HPLC system and a Waters 2487 UV wavelength detector (Waters; Milford, MA, USA) were used. Separation was performed on a Zorbax SB-C18 column (150 × 4.6 mm inner diameter, 3.5-µm particle size; Agilent Technologies; Santa Clara, CA, USA), with a column temperature of 55 °C. The mobile phase consisted of methanol, water, and acetonitrile (50:23:27, v/v). The flow rate was 1 mL/min, and the total run time was 15 min per sample. The wavelength was set at 277 nm, and the detector signals were recorded using Millennium 32 software (Waters; Milford, MA, USA). The injection volume was 90 µL, and the retention times for everolimus and the internal standard of ascomysin were 10.5 and 7.5 min, respectively (Fig. S1). The amount of everolimus taken up into cells was calculated from its concentration in the organic layer.

### Measurement of glucose in culture medium and C2C12 myotube cells

The glucose concentrations were measured using a Glucose Colorimetric/Fluorometric Assay Kit (Bio Vision; Milpitas, CA, USA). Briefly, 20 µL of collected culture medium or 5 µL of aqueous layer were diluted with assay buffer. Glucose reaction mixture was added and incubated for 30 min. The optical density was measured at 570 nm using a Sunrise microplate reader (TECAN; Seestrasse, Mannedorf, Switzerland). The concentration ranges of the calibration curves were 0–36 ng/µL for the culture medium and 0–9.2 µg/mL for the aqueous layer.

### Measurement of metabolites in C2C12 myotube cells

A 750 µL aliquot of the aqueous layer of C2C12 cell extract was passed through an ultrafiltration membrane (4 °C, 9100×*g* for approximately 180 min). The filtrate was evaporated during centrifugation under reduced pressure. The residue was reconstituted in 25 µL of water solution containing Internal Standard Solution-3 and transferred to autosampler vials. An Agilent 1600 CE System and an Agilent 6210 TOFMS System were used for measurement of anionic metabolites, and an Agilent 7100 CE System and Agilent 6530 Accurate-Mass Q-TOF LC/MS System were used for measurement of cationic metabolites (Agilent Technologies, Santa Clara, CA, USA). All metabolites were analyzed using a fuzed silica capillary (50 μm i.d.; 80 cm total length). Anion Buffer Solution (HMT; Tsuruoka, Yamagata, Japan) was used for anionic analysis and Cation Buffer Solution (HMT; Tsuruoka, Yamagata, Japan) was used for cationic analysis. In the anionic analysis, the sample was injected at a pressure of 50 mbar for 25 s. The applied voltage was set at 30 kV. Electrospray ionization–mass spectrometry (ESI–MS) was performed in the negative-ion mode. The cationic metabolites samples were injected at a pressure of 50 mbar for 10 s. The applied voltage was set at 27 kV. ESI–MS was then performed in the positive-ion mode. Both spectrometers were scanned from mass-to-charge (m/z) 50 to 1000. To obtain peak information, including m/z, migration time (MT), and peak area, peaks detected by CE-TOFMS were extracted using Master Hands automatic integration software (Keio University, Japan). The peaks were annotated with putative metabolites from the HMT metabolite database on the basis of their MT in CE and m/z values determined by TOFMS. The tolerance range for the peak annotation was configured at 0.5 min for MT and 50 parts per million for m/z. The identification and quantification of each metabolite was performed according to a previously reported method with slight modification (Soga et al. 2003). In addition, concentrations of the targeted metabolites were calculated by normalizing the peak area of each metabolite to the area of the Internal Standard Solution-1 and counted cell numbers.

### RNA extraction and real-time RT-PCR

Differentiated C2C12 cells were prepared for RT reaction and real-time RT-PCR using a TaqMan^®^ Gene Expression Cell-to-CT™ Kit and TaqMan^®^ gene expression assays according to the manufacturer’s protocol (Thermo Fisher Scientific; Waltham, MA, USA). Assay IDs of TaqMan probes are presented in Table 1. A Gene Amp PCR system 9700 (Thermo Fisher Scientific; Waltham, MA, USA) was used for RT reaction, and a 7900HT Fast Real-Time PCR System (Thermo Fisher Scientific) was used for real-time RT-PCR analysis. The reactions were run in 384-well plates using the following program according to the manufacturer’s protocol: 50 °C for 2 min followed by 95 °C for 10 min, 40 cycles at 95 °C for 15 s, and 60 °C for 1 min. All experiments were performed in triplicate. The data were normalized to β-actin (*Actb*). The relative quantities of mRNA for each sample were calculated using the comparative delta–delta cycle threshold method.

**Table 1:** List of genes measured by real-time RT-PCR in this study. TaqMan^®^ gene expression assays. The following TaqMan probes (Thermo Fisher Scientific; Waltham, MA, USA) were used in our study

Gene name (Assay ID)		
Aco1 (Mm00801417_m1)	Akt1 (Mm01331626_m1)	Akt2 (Mm02026778_g1)
Akt3 (Mm00442194_m1)	Aldoa (Mm00833172_g1)	Cs (Mm00466043_m1)
Dlst (Mm00513470_m1)	Eno1 (Mm01619597_g1)	Fh1 (Mm01321349_m1)
G6pdx (Mm00656735_g1)	Gapdh (Mm99999915_g1)	Gpi1 (Mm01962484_u1)
Hif1α (Mm00468869_m1)	Hk1 (Mm00439344_m1)	Idh1 (Mm00516030_m1)
Ldha (Mm01612132_g1)	Mdh1 (Mm00485106_m1)	Mtor (Mm00444968_m2)
Myc (Mm00487804_m1)	Ogdh (Mm00803119_m1)	Pck1 (Mm01247058_m1)
Pcx (Mm00500992_m1)	Pdha1 (Mm00468675_m1)	Pfkm (Mm01309576_m1)
Pgd (Mm00503037_m1)	Pgk1 (Mm00435617_m1)	Pgls (Mm00452598_m1)
Pkm (Mm00834102_g1)	Prkaa1 (Mm01296700_m1)	Prkaa2 (Mm01264789_m1)
Prkab1 (Mm01201921_m1)	Prkab2 (Mm01257133_m1)	Prkag1 (Mm00450298_g1)
Prkag2 (Mm00513977_m1)	Prkag3 (Mm00463997_m1)	Rpia (Mm00485790_m1)
Sdha (Mm01352363_m1)	Slc2a1 (Mm00441480_m1)	Slc2a4 (Mm01245502_m1)
Suclg2 (Mm01182162_g1)	Taldo1 (Mm00807080_g1)	Tkt (Mm00447559_m1)
Tpi1 (Mm00833691_g1)		

Reference gene: Actb (Mm00607939_s1)

### Western blot analysis

Cells were lysed using RIPA buffer (Wako Pure Chemical Industries, Ltd., 50 mmol/L Tris–HCl [pH8.0], 150 mmol/L sodium chloride, 0.5% (w/v) sodium deoxycholate, 0.1% (w/v) sodium dodecyl sulfate, 1.0% (w/v) NP-40 substitute), with protease inhibitor cocktail (NACALAI TESQUE, Inc., Kyoto-shi, Kyoto, Japan). After centrifugation at 15,000×*g* for 10 min at 4 °C to remove cellular debris, protein concentrations were determined by the Pierce BCA protein assay (Thermo Fisher Scientific Inc.). Proteins of interest were detected using 20 µg of whole cell lysates. Cell lysates were separated by sodium dodecyl sulfate polyacryamide gel electrophoresis. The separated proteins were transferred to polyvinylidene difluoride membranes (IPVH00010; Merck Millipore, Darmstadt, Germany) using a Trans-Blot^®^ SD Semi-Dry Transfer Cell (Bio-Rad Laboratories, Inc., Hercules, CA, USA). After blocking, membranes were incubated with each antibody followed by incubation with horseradish peroxidase-conjugated secondary antibodies (GE Healthcare, Little Chalfont, Buckinghamshire, UK). The dilutions of the anti-HK1, anti-PKM1/2, anti-PKM2, anti-LDHA, anti-PFKM, anti-PGD, and anti-TALDO antibodies were 1:1000, and the anti-GLUT1 and anti-γ-tubulin antibodies were 1:2000. Blots were developed using an Amersham ECL Prime Western Blotting Detection Reagent according to the manufacturer’s instructions (GE Healthcare). Protein bands were visualized using an LAS 4000 mini imaging system (GE Healthcare). γ-Tubulin was used as a loading control.

### Assays of enzyme activities

Enzyme activities were assayed using HK colorimetric assay kit, lactate dehydrogenase (LDH) activity assay kit, phosphofructokinase (PFK) activity colorimetric assay kit, pyruvate kinase activity assay kit (Sigma) and 6-PGD activity colorimetric assay kit (BioVision) according to the manufacturers’ protocols. The optical density was measured using a Sunrise microplate reader (TECAN; Seestrasse, Mannedorf, Switzerland).

### Statistical analysis

Experimental results were analyzed using a Student’s *t*-test. A p-value <0.05 was considered statistically significant.

## Results

### Diminished glucose consumption in the presence of everolimus

**Fig. 1:**
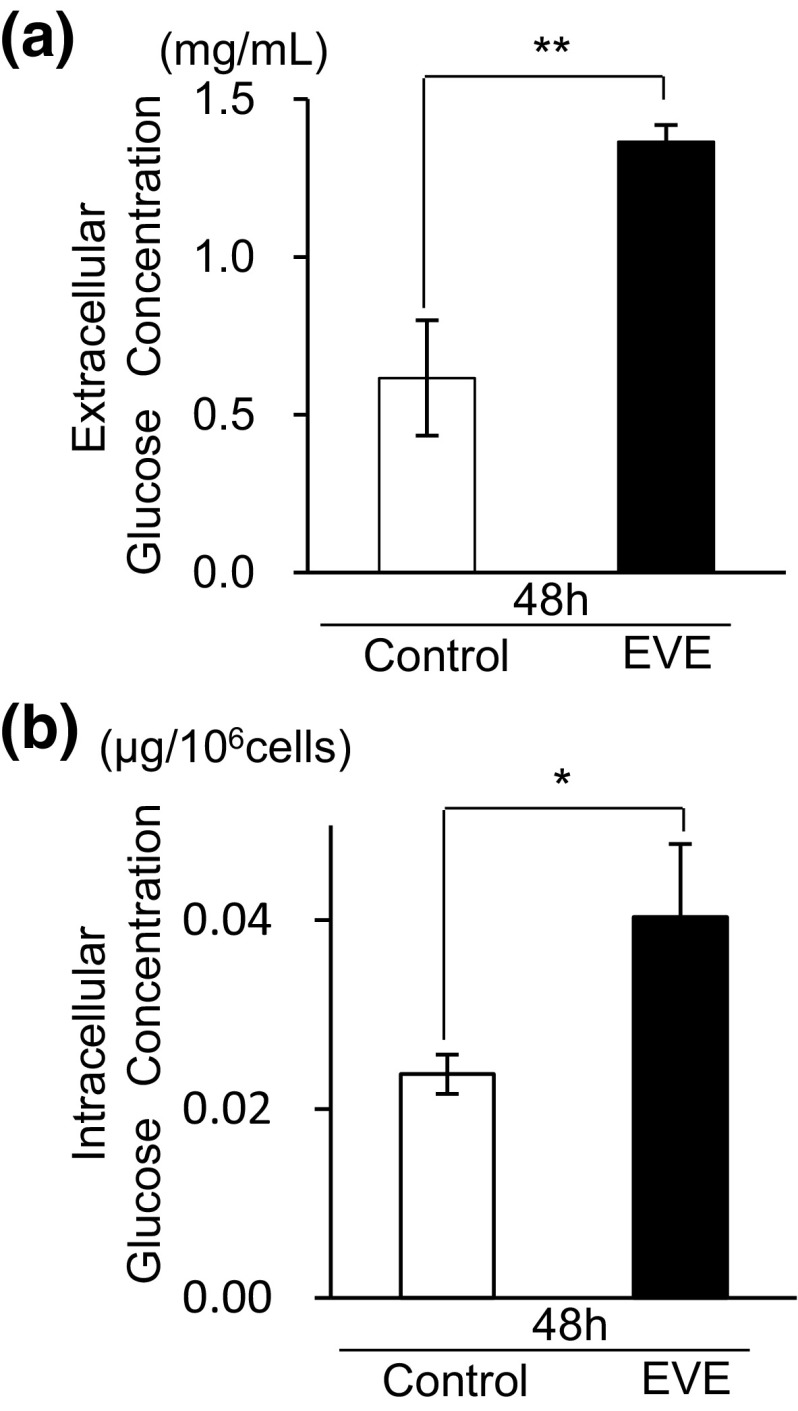
Changes in extracellular and intracellular glucose concentrations. At 0 h, 500 ng/mL of everolimus was added to the cultured media (EVE). **a** The cultured media were collected for measurement of extracellular glucose at 48 h. The original (0 h) concentration of cultured media was 4.5 mg/mL. The *open bar* represents the glucose concentration of the 48 h control sample and the *filled bar* denotes a sample exposed to everolimus for 48 h. Data represent the mean ± S.D. (n = 3). Statistically significant difference between EVE and control: **p < 0.01 (Student’s *t*-test). **b** Intracellular glucose concentration at 48 h. *Open bars* represent 48 h control cells and *filled bars* denote 48 h everolimus-exposed cells. Data represent the mean ± S.D. (n = 3). Statistically significant difference between EVE and control: *p < 0.05 (Student’s *t*-test)

Uptake of everolimus by cells was 5.87 ± 0.06 ng/10^6^ cells when exposed for 48 h. After 48 h, glucose concentrations of the culture medium, with or without everolimus, decreased from the baseline level (4.5 mg/mL) to 0.62 ± 0.05 mg/mL in the control cells and 1.37 ± 0.06 mg/mL in the everolimus-exposed cells (Fig. 1a). The glucose concentration of the everolimus-exposed cell was two times higher than the control cells (**p < 0.01). The results showed that the skeletal muscle cell model resembled hyperglycemia.

### Intracellular glycometabolism

Intracellular glucose levels also decreased during 48 h in both the control and everolimus-exposed cells. Furthermore, the glucose levels of the everolimus-exposed cells were higher than that of the control cells (Fig. 1b).

**Fig. 2:**
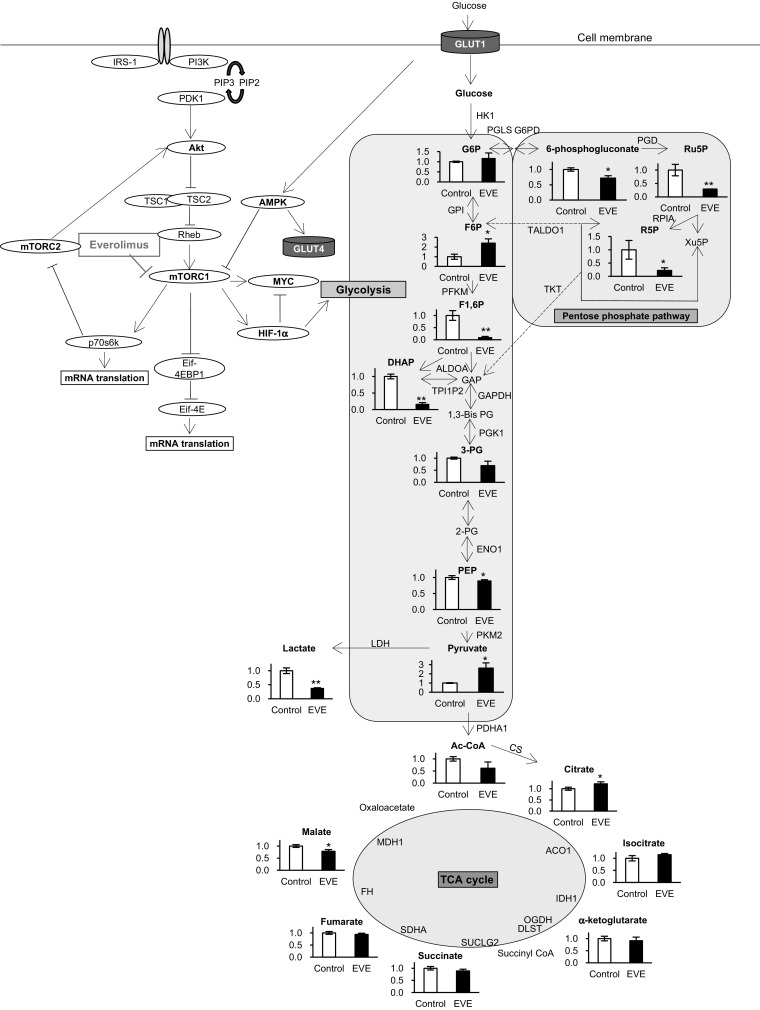
Metabolic profiling of intracellular glycometabolism. Changes in metabolite levels after 48 h exposure to everolimus (*closed bars*, EVE) were compared with those without everolimus at 48 h (Control). The data represent the mean ± S.D. (n = 3). Statistically significant differences between the EVE and control: *p < 0.05; **p < 0.01 (Student’s *t*-test). Abbreviations: *G6P* glucose 6-phosphate, *F6P* fructose 6-phosphate, *F1,6P* fructose 1,6-bisphosphate, *GAP* glyceraldehyde phosphate, *DHAP* dihydroxyacetone phosphate, *1,3-Bis-PG* 1,3-Bis phosphoglycerate, *3PG* 3-phosphoglycerate, *2PG* 2-phosphoglycerate, *PEP* phosphoenolpyruvate, *Ac-CoA* acetyl CoA, *Ru5P* ribulose 5-phosphate, *R5P* ribose 5-phosphate, *Xu5P* xylulose 5-phosphate, *mTOR* mammalian target of rapamycin, *HIF1a* hypoxia-inducible factor-1α, *GLUT* glucose transporter, *HK1* hexokinase-1, *GPI* glucose-6-phosphate isomerase, *PFKM* 6-phosphofructokinase, *ALDOA* aldolase, *TPI1P2* triosephosphate isomerase, *GAPDH* glyceraldehyde 3-phosphate dehydrogenase, *PGK1* phosphoglycerate kinase-1, *ENO1* enolase-1, *PKM2* pyruvate kinase-2, *LDHA*_lactate dehydrogenase A, *PDHA1* pyruvate dehydrogenase α1, *G6PD* 6-phosphate dehydrogenase, *PGD* 6-phosphogluconate dehydrogenase, *RPIA* ribose-5-phosphate isomerase A, *TKT* transketolase, *TALDO1* transaldolase-1, *CS* citrate synthase, *ACO1* aconitase-1, *IDH1* isocitrate dehydrogenase-1, *OGDH* α-ketoglutarate dehydrogenase, *DLST* dihydrolipoamide succinyltransferase, *SUCLG2* succinyl-CoA ligase, *SDHA* succinate dehydrogenase A, *FH* fumarate hydratase, *MDH1* malate dehydrogenase-1

**Fig. 3:**
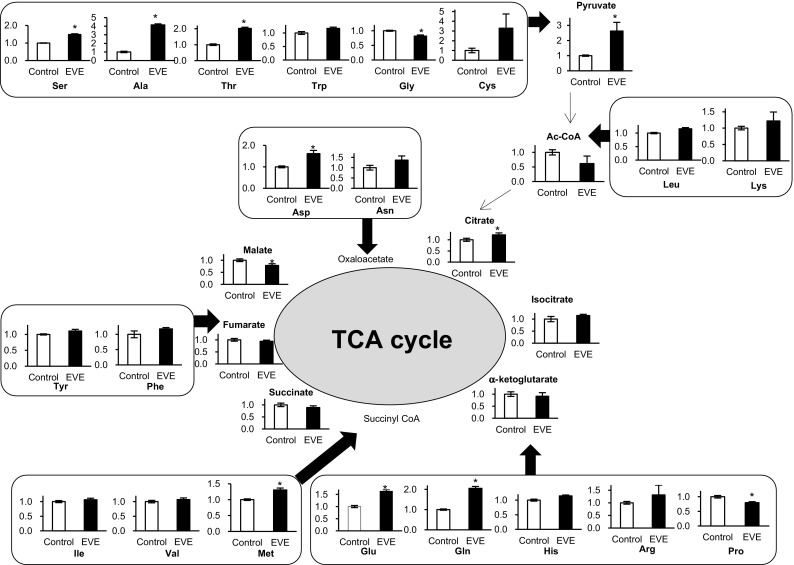
Metabolic profiling of intracellular amino acid and TCA cycle. Changes in metabolite levels after 48 h exposure to everolimus (*closed bars*, EVE) were compared to those without everolimus at 48 h (Control). The data represent mean ± S.D. (n = 3). Statistically significant difference between EVE and control: *p < 0.05; **p < 0.01 (Student’s *t*-test). Abbreviations: *Ala* alanine, *Arg* arginine, *Asn* asparagine, *Asp* aspartic acid, *Cys* cysteine, *Glu* glutamic acid, *Gln* glutamine, *Gly* glycine, *His* histidine, *Ile* isoleucine, *Leu* leucine, *Lys* lysine, *Met* methionine, *Phe* phenylalanine, *Pro* proline, *Ser* serine, *Thr* threonine, *Trp* tryptophan, *Tyr* tyrosine, *Val* valine

Glucose is taken up by cells and metabolized via a series of pathways. We examined the three major metabolic pathways: glycolysis, the TCA cycle, and the PPP. The key metabolites in these pathways were measured. Figure 2 shows the changes in these metabolites compared with those of the controls. In glycolysis, levels of fructose 1,6-bisphosphate (F1,6P), dihydroxyacetone phosphate, phosphoenolpyruvate, and lactate in the everolimus-exposed cells were lower, and fructose 6-phosphate (F6P) and pyruvate were higher than those of the control. There were no remarkable differences in the metabolites of the TCA cycle between the two groups. We also measured 20 amino acids linked to the TCA cycle (Fig. 3) and found their concentrations were changed by everolimus, with some decreased (glycine, proline) and others were elevated (serine, alanine, aspartic acid, threonine, cysteine, glutamic acid, glutamine, and methionine). All of the PPP metabolites, including 6-phosphogluconate, ribulose 5-phosphate (Ru5P), and ribose 5-phosphate (R5P), were significantly reduced by everolimus (Fig. 2). The results showed that everolimus reduced the activity of most of the glucose-related metabolic pathways.

### mRNA abundance of glycometabolic enzymes

**Fig. 4:**
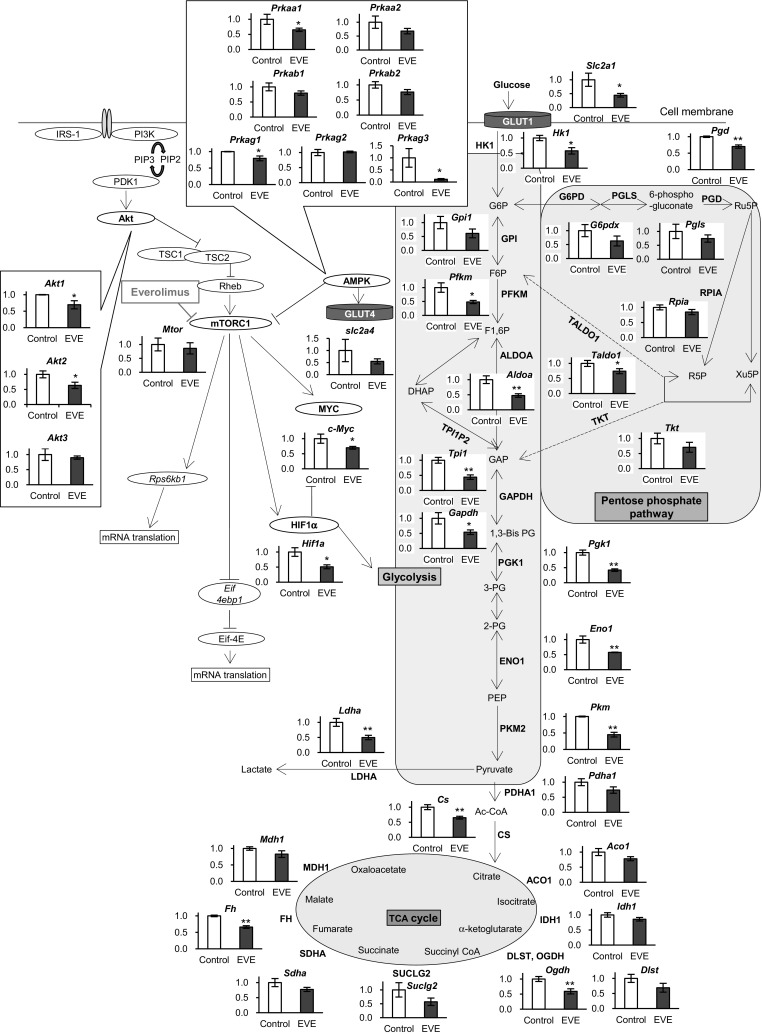
mRNA expression of enzymes and factors involved in glycometabolism. Changes in expression levels after 48 h exposure to everolimus (*closed bars*, EVE) were compared with those without everolimus at 48 h (Control). The data represent the mean ± S.D. (n = 3). Statistically significant difference between EVE and control: *p < 0.05; **p < 0.01 (Student’s *t*-test). Abbreviations are the same as Fig. 2

To investigate the mechanism of everolimus-related reduction of the glycometabolic activity of cells, we measured mRNA abundance of GLUT1 and GLUT4, enzymes of glycometabolism, and mTOR signal-related proteins, using real-time RT-PCR. After 48 h exposure to everolimus, the expression of the genes decreased. The mRNA of GLUT1 (*Slc2a1*) was decreased significantly by everolimus exposure while GLUT4 (*Slc2a4*) showed no significant change (Fig. 4). In the glycolysis pathway, mRNA abundance of five enzymes, including aldolase (*Aldoa*), triosephosphate isomerase (*Tpi1*), glyceraldehyde 3-phosphate dehydrogenase (*Gapdh*), phosphoglycerate kinase-1 (*Pgk1*), and enolase-1 (*Eno1*), as well as mRNA abundance of four rate-limiting enzymes, including hexokinase-1 (*Hk-1*), PFKM (*Pfkm*), PKM2 (*Pkm*), and LDHA (*Ldha*), decreased with exposure to everolimus (Fig. 4). Citrate synthase (*Cs*), which connects to the glycolysis pathway in the TCA cycle, and α-ketoglutarate dehydrogenase (*Ogdh*) and fumarate hydratase (*Fh*) in the TCA cycle, also decreased. mRNA abundance of PPP, PGD (*Pgd*), and TALDO1 (*Taldo1*) also decreased (Fig. 4).

### mTOR signaling pathway

Although everolimus did not affect mTOR (*Mtor*) mRNA abundance itself (Fig. 4), mRNA abundance of HIF1α (*Hif1a*) and c-Myc (*Myc*), which function as transcription factors downstream of mTOR, were lowered by everolimus. Interestingly, upstream of the mTOR pathway, mRNA abundance of Akt1 (*Akt1*) and Akt2 (*Akt2*) were also decreased (Fig. 4). Furthermore, 5′-monophosphate protein kinase (AMPK) mRNA abundance was changed by everolimus. Everolimus significantly suppressed AMPKα1 (*Prkaa1*), γ1 (*Prkag1*), and γ 3 (*Prkag3*) (Fig. 4).

### Everolimus reduced protein expression and enzyme activity in the glycometabolic pathway

**Fig. 5:**
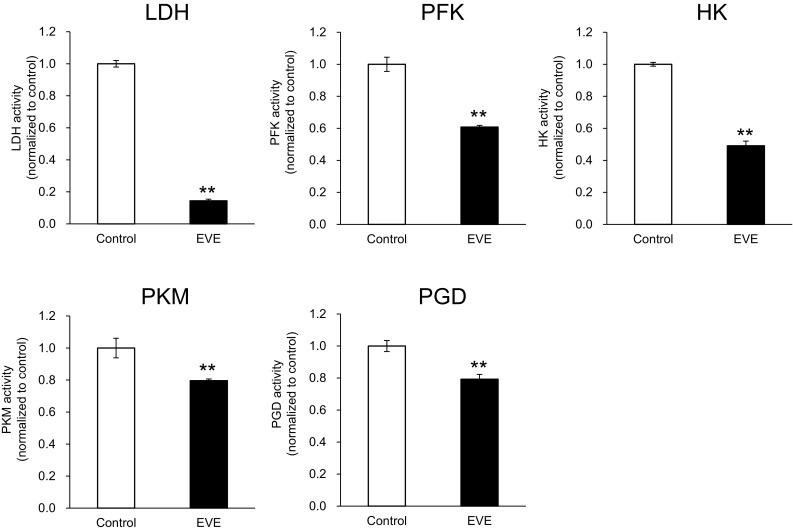
Activity for key enzymes involved in glycolysis and pentose phosphate pathway. Changes in enzyme activity after 48 h exposure to everolimus (*closed bars*, EVE) were compared with those without everolimus at 48 h (Control). The data represent the mean ± S.D. (n = 3). Statistically significant difference between EVE and control: **p < 0.01 (Student’s *t*-test). Abbreviations: *LDH* lactate dehydrogenase, *PFK* phosphofructokinase, *HK* hexokinase, *PKM* pyruvate kinase, *PGD* 6-phosphogluconate dehydrogenase. The other abbreviations are the same as Fig. 2

Based on the results of the gene expression assay, we performed Western blots for glycometabolic enzymes including GLUT1, HK1, PFKM, PKM (1/2, 2), LDHA, PGD, and TALDO1 which showed significant changes. Protein expression was slightly decreased by everolimus, although the change was not statistically significant between everolimus-exposed and control cells (Fig. S2). We also measured the enzyme activities of HK, PFK, PKM, LDH, and PGD, which were significantly reduced by exposure to everolimus (Fig. 5).

## Discussion and conclusions

Glycometabolic changes induced by mTOR inhibitors have been examined previously (Beretta et al. 1996; Sun et al. 2011). Because mTOR proteins are located downstream of the insulin signal, most studies have focused on insulin secretion or insulin resistance (Lamming et al. 2012). For example, a study on *Tsc2* in pancreatic β-cell-deficient mice showing abnormal activation of the mTOR signal in pancreatic cells demonstrated an association between mTOR and insulin secretion (Rachdi et al. 2008). Other studies have shown that rapamycin-induced mTOR inhibition improves insulin resistance, likely because the relationship between mTOR activity and insulin sensitivity follows a U-shape (Laplante and Sabatini 2012; Leontieva et al. 2014). Previous publications have demonstrated that an insulin-dependent mechanism plays a role in hyperglycemia associated with mTOR inhibition. However, mTOR proteins are present throughout the body, and everolimus is selective for the site of action but does not have a specific distribution; therefore, we studied another potential mechanism of hyperglycemia that could be insulin-independent glucose metabolism in peripheral cells.

It is known that mTOR is related to GLUT expression (Kohn et al. 1996; Taha et al. 1999). Our findings of an increase in extracellular glucose level (Fig. 1a) and a decrease in GLUT1 mRNA abundance (Fig. 4) suggest that glucose uptake was decreased by everolimus because of diminished GLUT1 expression. Actually, the result of GLUT1 protein expression assay showed everolimus slightly decreased GLUT1 (Fig. S2). However, the intracellular glucose concentration in everolimus-exposed cells was higher than that in control cells (Fig. 1b). These findings suggest that everolimus might not only have suppressed expression of glucose transporters but might also have depressed glucose utilization in cells.

CE-TOFMS measurements indicated that the levels of several metabolites were altered by everolimus. Specifically, F6P and pyruvate levels were significantly higher than those of controls (Fig. 2). F6P is transformed to F1,6P by PFKM. In our study, F6P increased and F1,6P decreased after exposure to everolimus (Fig. 2), and PFKM mRNA (*Pfkm*) expression was suppressed in everolimus-exposed cells (Fig. 4). PFK activity was significantly reduced by everolimus (Fig. 5), implying diminished activity of the rate-limiting reaction in glycolysis. Additionally, everolimus increased pyruvate and decreased lactate relative to the concentrations in the control cells (Fig. 2). LDHA and citrate synthase (CS) mRNA expression was inhibited by everolimus. LDHA relates to irreversible lactate production and is located between pyruvate and lactate, and CS transforms acetyl CoA and oxaloacetate to citrate (Fig. 4). In our study, LDH activity was decreased significantly by everolimus (Fig. 5). Glycometabolism was likely stagnated because of reduced mRNA abundance of rate-limiting enzymes. Amino acids also may cause fluctuations in pyruvate levels. The results showed that the amino acids (serine, alanine, threonine, and cysteine) related to supply of pyruvate were greatly increased by everolimus (Fig. 3). This phenomenon was potentially caused by protein synthesis and proteolysis balance in skeletal muscle cells or autophagy (Tan and Miyamoto 2016).

In the PPP, 6-phosphogluconate, Ru5P, and R5P were decreased significantly by everolimus (Fig. 2). Transformation of G6P to Ru5P is an irreversible reaction and is triggered by G6PD, PGLS, and PGD. We observed decreased expression of *Pgd* and *Taldo1* (Fig. 4), consistent with the results of a previous study in which expression of *Pgd* and *Taldo1* was induced by mTORC1 and decreased by rapamycin (Duvel et al. 2010). Additionally, our result showed that PGD activity was lowered by everolimus after 48-h exposure (Fig. 5). Therefore, the PPP was inhibited by everolimus via decreased expression and activity of *Pgd* and *Taldo1*.

There were no significant changes to the metabolites and genes of the TCA cycle (Figs. 2, 4). Interestingly, the metabolites of the glycolysis pathway were decreased by everolimus, but stagnation of glycolysis did not affect the TCA cycle. At the same time, some amino acids were increased after exposure to everolimus. We speculated that the reduced supply of glycolytic metabolites to the TCA cycle was compensated for by the increased concentration of amino acids (Fig. 3).

*Hif1a* and *Myc* were decreased by everolimus (Fig. 4). Expression of HIF1α is enhanced under hypoxia and reduces c-Myc expression through inhibition of the activity of mitochondria (Dang et al. 2008; Hay and Sonenberg 2004; Yeung et al. 2008). Previous studies showed that HIF1α and c-Myc activated glycolysis (Cheng et al. 2014; Majmundar et al. 2010; Masui et al. 2013, 2014; Yeung et al. 2008). The function of mTORC1 in muscles is often referred to as myoblast differentiation, but its regulation of glycolysis via HIF1α and c-Myc has not been proven (Cornu et al. 2013; Willett et al. 2009). In the present study, suppression of HIF1α and c-Myc mRNA abundance was observed in parallel with decreased activity of glycolysis caused by inhibition of mTORC1 by everolimus. Inhibition of glycolytic function could lead to a decrease in intra- and extracellular glucose consumption.

We also examined Akt and AMPK upstream of mTOR. After 48-h everolimus exposure, AMPKα1, γ1, and γ3 mRNA decreased significantly (Fig. 4). These isoforms are present in skeletal muscle cells. AMPKγ3 is expressed in particular in skeletal muscle (Cheung et al. 2000), and it is known that pharmacological and physiological AMPK activity is inhibited by eliminating AMPKγ3 (Barnes et al. 2005a, b; Canto et al. 2010). The present result indicates that everolimus reduces AMPK function by lowering AMPKγ3. *Akt1* and *Akt2* were also decreased by everolimus (Fig. 4). The Akt1 protein is expressed throughout the body, and Akt2 is expressed in insulin-sensitive peripheral tissue, such as muscle (Koseoglu et al. 2007). Our results showed that everolimus affected mRNA abundance downstream as well as upstream of mTOR. Further analysis is required to clarify the roles of AMPK and Akt on hyperglycemia.

In this study, we focused on the three major glucose metabolic pathways and showed that everolimus reduced glucose metabolism in C2C12 skeletal muscle cells. Everolimus lowered mRNA abundance of the glycolysis enzymes; especially expression and activity of rate-limiting enzymes, which were significantly reduced. Furthermore, everolimus decreased the PPP, whereas no change was observed in the TCA cycle. One limitation of the present study is that the metabolomic data only showed static changes of pathways after 48 h everolimus exposure. Further flux analysis is required to clearly elucidate the perturbations to glucose metabolism.

The series of experiments were conducted to obtain better understanding of transcription, translation, enzymatic activity and metabolic consequences. We showed that everolimus treatment perturbed the intracellular metabolism and decreased mRNA abundance. Its influence on the protein expression (HK, PFK, PKM, LDH and PGD) was slight but their enzymatic activities were significantly reduced. Since there was a gap between the transcription and protein expression, measurement of enzymatic activity was important to fill the gap. The observed discrepancy is interesting since everolimus treatment did not equally impact transcription, protein expression and enzymatic activity. The reason for this discrepancy is unknown, but it can be speculated that the observed phenomena resulted from systematic change in complex multiple pathways caused by direct and indirect effects of everolimus rather than sole inhibition of mTORC1. Another possibility is that a different amount of time is needed for changes in the expression or activity. We measured transcription, protein expression and enzymatic activity after the same exposure time (48 h), therefore further analysis of different time courses needs to be performed in the future.

In conclusion, we report a metabolomic analysis indicating that everolimus impairs glucose metabolism in muscle cells by lowering the activity of glycolysis and the PPP, which implies a potential mechanism for everolimus-associated hyperglycemia observed clinically.

## Electronic supplementary material

Below is the link to the electronic supplementary material.

**Supplemental Figure S1**: Representative chromatogram of everolimus and ascomycin (as the internal standard) for a sample prepared from C2C12 cell extract. Retention times for ascomycin and everolimus were 7.4 and 10.5 min, respectively. (PPTX 163 KB)

**Supplemental Figure S2**: Results of the Western blot analysis. Protein expression for GLUT-1 and key enzymes was evaluated by Western blotting analysis. The changes in protein expression after 48 h exposure to everolimus (closed bars, EVE) were compared with those without everolimus at 48 h (Control). The data represent the mean ± S.D. (n = 3). Statistically significant difference between EVE and control: *p < 0.05 (Student’s *t*-test). Abbreviations are the same as Fig. 2. (PPTX 154 KB)
