# Supplementary figures and images for: Effect of everolimus on the glucose metabolic pathway in mouse skeletal muscle cells (C2C12)
Source: Metabolomics. 2017 Jul 7;13(8):98. doi: 10.1007/s11306-017-1236-5 (PMC5501892; doi:10.1007/s11306-017-1236-5)

## Slide 1
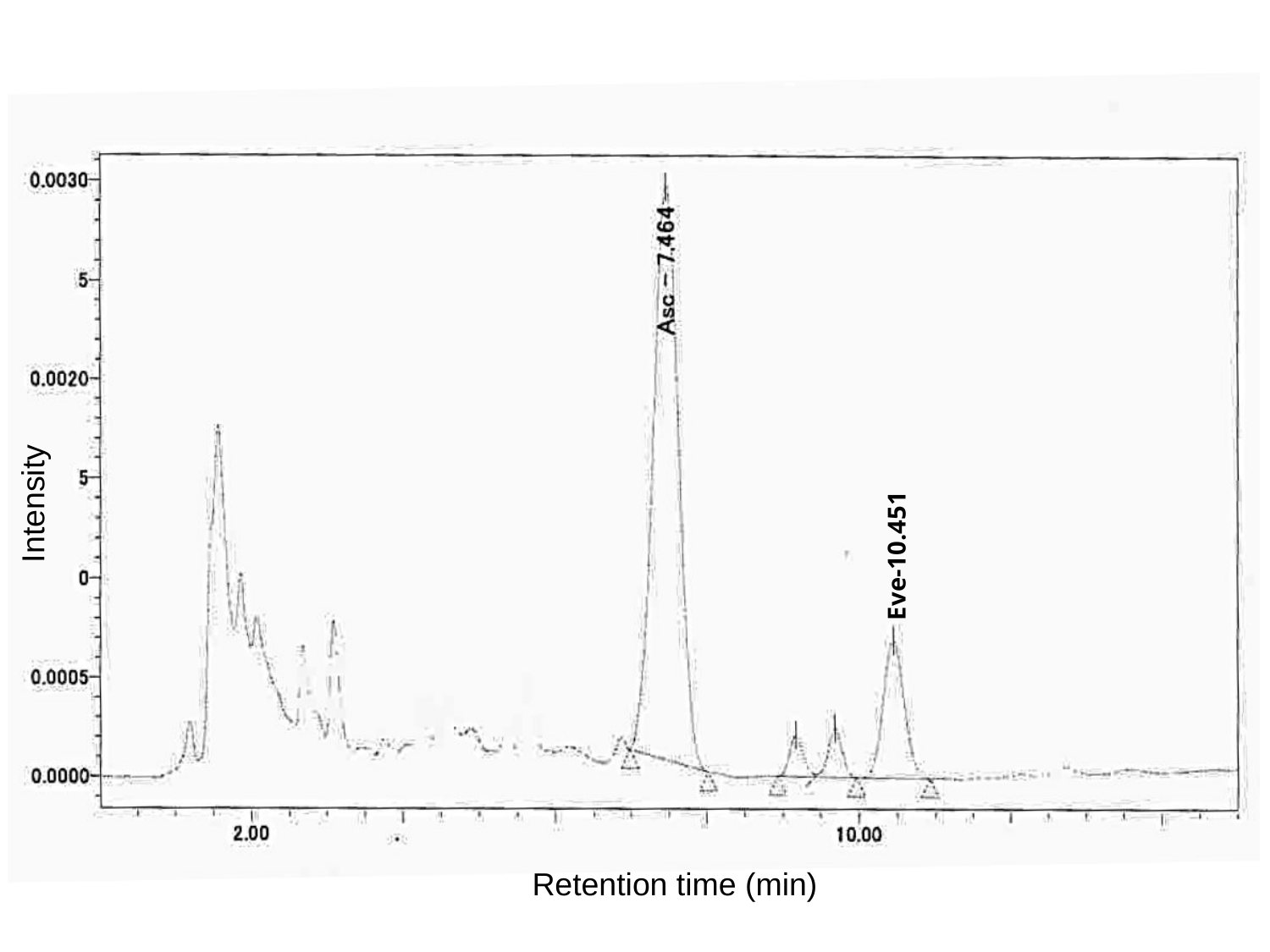

Intensity
Eve-10.451
Retention time (min)

Supplement: Supplementary file 1 — Supplemental Figure S1: Representative chromatogram of everolimus and ascomycin (as the internal standard) for a sample prepared from C2C12 cell extract. Retention times for ascomycin and everolimus were 7.4 and 10.5 min, respectively. (PPTX 163 KB) [file 11306_2017_1236_MOESM1_ESM.pptx]
