# Supplementary material for: Effect of everolimus on the glucose metabolic pathway in mouse skeletal muscle cells (C2C12)
Source: Metabolomics. 2017 Jul 7;13(8):98. doi: 10.1007/s11306-017-1236-5 (PMC5501892; doi:10.1007/s11306-017-1236-5)
Supplement: Supplementary file 2 — Supplemental Figure S2: Results of the Western blot analysis. Protein expression for GLUT-1 and key enzymes was evaluated by Western blotting analysis. The changes in protein expression after 48 h exposure to everolimus (closed bars, EVE) were compared with those without everolimus at 48 h (Control). The data represent the mean ± S.D. (n = 3). Statistically significant difference between EVE and control: *p < 0.05 (Student’s t-test). Abbreviations are the same as Fig. 2. (PPTX 154 KB) [file 11306_2017_1236_MOESM2_ESM.pptx]

## Slide 1
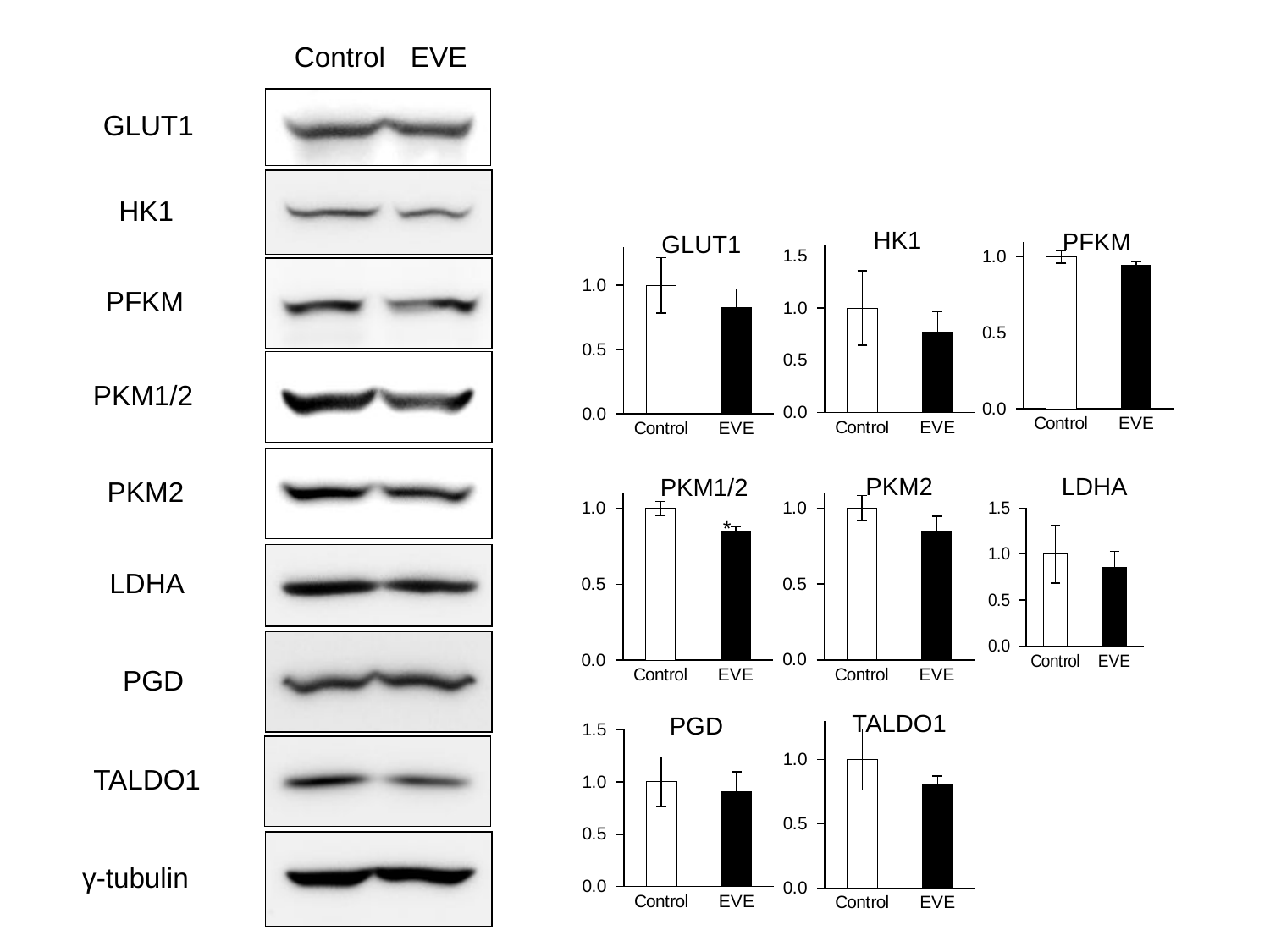

Control
EVE
GLUT1
HK1
HK1
PFKM
GLUT1
### Chart
| Category | |
|---|---|
| Control | 1.0 |
| EVE | 0.9466854037417979 |
### Chart
| Category | |
|---|---|
| Control | 1.0 |
| EVE | 0.7711377770368697 |
### Chart
| Category | |
|---|---|
| Control | 1.0 |
| EVE | 0.8288132974475337 |
PFKM
PKM1/2
PKM2
LDHA
PKM1/2
PKM2
### Chart
| Category | |
|---|---|
| Control | 1.0 |
| EVE | 0.8528020883192711 |
### Chart
| Category | |
|---|---|
| Control | 1.0 |
| EVE | 0.8480207401319699 |
### Chart
| Category | |
|---|---|
| Control | 1.0 |
| EVE | 0.8500308721932881 |*
LDHA
PGD
TALDO1
PGD
### Chart
| Category | |
|---|---|
| Control | 1.0 |
| EVE | 0.9083458215771035 |
### Chart
| Category | |
|---|---|
| Control | 1.0 |
| EVE | 0.8049691821799195 |
TALDO1
γ-tubulin
